# Interstitial deletion at 11q14.2-11q22.1 may cause severe learning difficulties, mental retardation and mild heart defects in 13-year old male

**DOI:** 10.1186/s13039-015-0175-y

**Published:** 2015-09-17

**Authors:** Ioannis Papoulidis, Vassilis Paspaliaris, Elisavet Siomou, Sandro Orru, Roberta Murru, Stavros Sifakis, Petros Nikolaidis, Antonios Garas, Sotirios Sotiriou, Loretta Thomaidis, Emmanouil Manolakos

**Affiliations:** Access to genome P.C., Clinical Laboratory Genetics, 33A Ethn. Antistaseos str, 55134 Thessaloniki, Greece; Department of Medical Genetics, University of Cagliari, Binaghi Hospital, Cagliari, Italy; Department of Obstetrics and Gynecology, University Hospital of Heraklion, Heraklion, Crete Greece; Embryoiatriki-genetiki Ltd, 49 Kifisias Ave., Athens, Greece; Department of Gynecology, Larissa Medical School, University of Thessaly, Larissa, Greece; Developmental Assessment Unit, Second Department of Paediatrics, P&A Kyriakou Children’s Hospital, University of Athens, 11527 Athens, Greece

## Abstract

Interstitial deletions of the long arm of chromosome 11 are rare, and they could be assumed as non-recurrent chromosomal rearrangements due to high variability of the size and the breakpoints of the deleted region. The exact region of the deletion was difficult to be determined before the use of molecular cytogenetic techniques such as array comparative genomic hybridization (aCGH). Here, a 13-year old boy with severe learning difficulties, mental retardation and mild heart defects is described. Conventional G-band karyotyping was performed and it is found that the patient is a carrier of a de novo interstitial deletion on the long arm of chromosome 11, involving 11q14 and 11q22 breakpoints. Further investigation, using aCGH, specified the deleted region to 11q14.2-11q22.1. There was a difficulty in correlating the genotype with the phenotype of the patient due to lack of similar cases in literature. More studies should be done in order to understand the genetic background that underlies the phenotypic differences observed in similar cases.

## Background

Terminal deletions of the long arm of chromosome 11 have been numerously described, and they are associated with Jacobsen syndrome (OMIM 147791) and characterized by thrombocytopenia, mental retardation, short stature, congenital heart defect, and characteristic facial dysmorphism [1, 2]. On the contrary, interstitial deletions of the long arm of chromosome 11 are less common and often not fine-mapped, due to the similarity between band patterns (11q14 and 11q22) when conventional karyotype is performed [3, 4]. So far, approximately 30 cases of 11q interstitial deletions have been reported [3–24]. Nevertheless, due to high variability of size and position of the deleted regions, phenotype-genotype correlations have been hard to evaluate due to the wide range of phenotypic features, ranging from normal to severe conditions including developmental delay/mental retardation, facial dysmorphisms and other medical implications. Moreover, before the introduction of molecular cytogenetic approaches, the resolution efficiency provided by conventional karyotype analysis jointly with the symmetric 11q banding pattern [3, 4], limited the accuracy of identification of breakpoints and precise deleted genomic regions.

### Case presentation

The patient, a 13-year old boy, was the first and only child of unrelated healthy Caucasian parents. He was born by cesarean section after a full term pregnancy. Birth weight was 2,800 g (10^th^ percentile), length 50 cm (50^th^ percentile) and head circumference (HC) 35.5 cm (50^th^ percentile). Neonatal and infancy periods were uneventful; nevertheless his motor development was delayed as he did not sit independently until the age of 15 months or walk unaided until the age of 22 months.

At the age of 5, language delay was observed and laboratory investigation was performed, including audiogram, biochemical and thyroid tests, which proved normal. He received speech therapy for a two-year period and his language difficulties resolved. He attended mainstream primary school with extra educational support and finished this level at the age of 12 years. At the age of 13 years he was referred for full developmental assessment because he was experiencing severe learning difficulties in secondary school. Upon physical examination, he was characterized as quite a sociable child, with mild dysmorphic facial features such as almond shaped eyes, hypertelorism, anteverted nostrils, and gothic palate. His weight at the time was 49 kg (40^th^ percentile), height 160 cm (60^th^ percentile) and HC 54 cm (25^th^ percentile). Upon neurological examination, he showed mild motor delay with severe clumsiness but without focal neurological signs. Ophthalmologic examination was normal. Heart auscultation revealed a mild systolic murmur.

Upon developmental evaluation, he was found to function at the mental level of a 10-year old. His cognitive abilities, according to the Wechsler Intelligence Scale for Children test (WISC III), were assessed as borderline, as his full-scale score was 75 with verbal score 75 and performance score 80.

Extensive laboratory investigation followed, including brain magnetic resonance imaging (MRI), electroencephalogram, kidney-liver- spleen ultrasound, bone age, biochemical tests, blood and urine amino acids, organic acids, very low fatty acids, thyroid function, ACTH, FSH, LH, DHEA-S, prolactin, estradiol e2, progesterone, testosterone, 17a-OH progesterone and IGF-1, all proved normal. Heart ultrasound showed mild mitral valve prolapsed.

## Material and methods

**Fig. 1:**
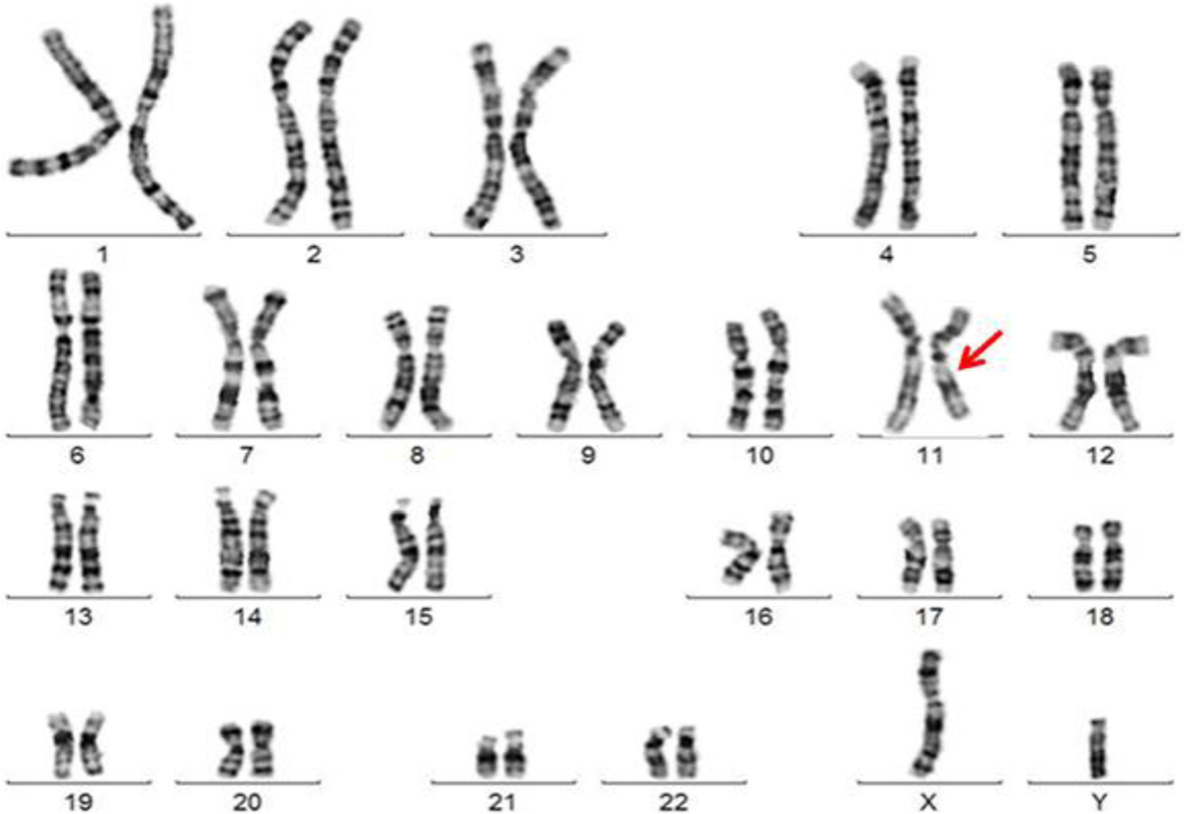
G-banding karyotype of the patient. It is illustrated the interstitial deletion on the long arm of chromosome 11

Metaphase chromosomes were obtained from phytohemagglutinin (PHA)-stimulated peripheral blood lymphocytes and high resolution (thymidine treatment) G-banding karyotype analysis was performed, using standard procedures. The conventional cytogenetic analysis revealed a de novo interstitial deletion on the long arm of chromosome 11, involving 11q14 and 11q22 breakpoints (Fig. 1).

To further investigate the specific finding, array-CGH was performed by hybridizing the sample against a male human reference commercial DNA sample (Promega biotech) using an array-CGH platform that includes 60000 oligonucleotides distributed across the entire genome (Agilent Technologies). The statistical test used as parameter to estimate the number of copies was ADAM-2 (provided by the DNA analytics software, Agilent Techn) with a window of 0.5 Mb, A=6. Only those copy number changes that affected at least 5 consecutive probes with identically oriented change were considered as Copy Number Variations (CNV). As a consequence, for the majority of the genome, the average genomic power of resolution of this analysis was 200 kilobases.

**Fig. 2:**
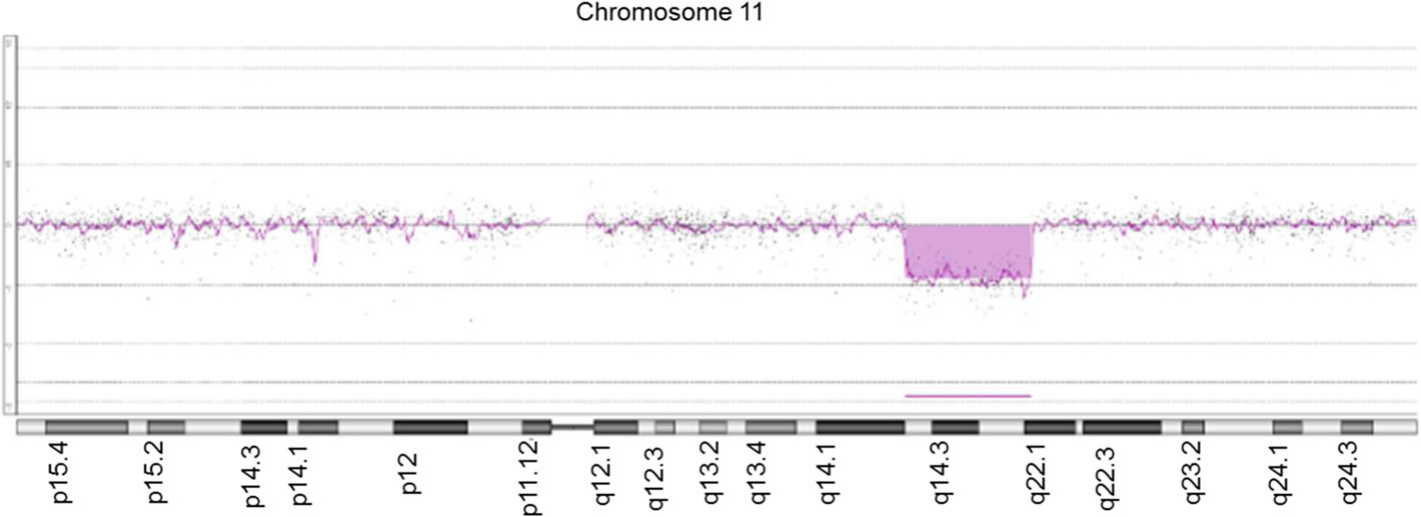
Ideogram of the deleted region as it is detected using array CGH. The interstitial deletion is at 11q14.2-11q22.1

**Fig. 3:**
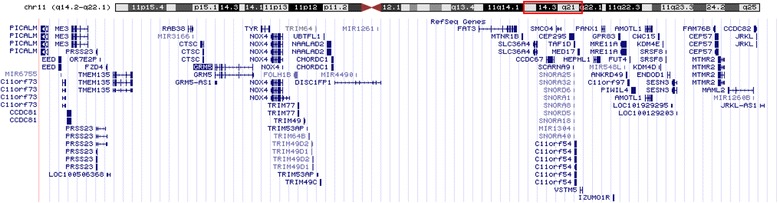
The 11q14.2 – 11q22.1 region is haploinsufficient to the current patient. The red box shows the region which is deleted. Below the chromosome ideogram are the transcripts of genes which are in the 11q14.2-11q22.1 region (http://genome.ucsc.edu/)

Array-CGH analyses detected an interstitial deletion spanning region 11q14.2-11q22.1, genomic coordinates chr11: 85,702,633-97,854,695 (Genomic coordinates are listed according to genomic build GRCh37/hg19). No additional pathogenetic Copy Number Variations (CNVs) were detected and thus the molecular karyotype was: arr 11q14.2q22.1 (85702633–97854695)x1 (Fig. 2). The specific deletion contains approximately 12,15 Mb of genomic material and includes 30 OMIM listed genes (Table 1 and Fig. 3).

**Table 1:** OMIM listed genes included in the 11q14.2-11q22.1 region

Gene	OMIM	Start	End	Cytogenetic region	Description	Protein function
PICALM	603025	85668485	58750108	11q14.2	Phosphatidylinositol binding clathrin assembly protein	Involved in cellular trafficking, regulation of endocytosis, and clathrin-mediated vesicle formation
EED	605984	85955815	85989781	11q14.2	Embryonic ectoderm development	Mediates repression of gene activity through histone deacetylation
*ME3*	604626	86152150	86383678	11q14.2	Malic enzyme 3, NADP(+)-dependent, mitochrondrial	Catalyzes the oxidative decarboxylation of malate to pyruvate using either NAD+ or NADP+ as a cofactor
*FZD4*	604579	86656721	86666433	11q14.2	Frizzled homolog 4 (Drosophila)	Receptor for Wnt proteins
*RAB38*	606281	87846431	87908599	11q14.2	RAB38, member RAS oncogene family	May be involved in melanosomal transport and docking
*CTSC*	602365	88026760	88070941	11q14.2	Cathepsin C	Lysosomal protease capable if removing dipeptides from the amino terminus of protein substrates
*GRM5*	604102	88237744	88796816	11q14.2	Glutamate receptor, metabotropic 5	Transduce signals from extra cellular transmitters to the inside of the cell by activating G proteins
TYR	606933	88911040	89028927	11q14.3	Tyrosinase (oculocutaneous albinismIA)	Conversion of tyrosine to melanin
*NOX4*	605261	89057521	89231363	11q14.3	NAPDH pxidase 4	may function as catalytic component of an endothelial NAPDH oxidase/may fulfill the function of oxygen sensor in the kidney
*FOLH1B*	609020	89392465	89431886	11q14.3	Folate hydrolase 1B	hydrolyzes beta-citrylglutamate/ found in the CNS during pre-perinatal periods of development in the testis in adult males
*TRIM49*	606124	89530823	89541743	11q14.3	Tripartite motif containing 49	Protein-protein interaction. Expressed mostly in testis
*NAAIAD2*	611636	89867818	89925779	11q14.3	N-acetylated alpha-linked acidic dipeptidase 2	NAALADase activity. Inactivate the peptide neurotransmitter N-acetylaspartylglutamate
*CHORDC1*	604353	89933597	89956532	11q14.3	Cysteine and histidine-rich domain (CHORD) containing 1	Function of the wildtype gene in nematode development
*FAT3*	612483	92085262	92629636	11q14.3	FAT tumor suppressor homolog 3 (Drosophila)	Cell adhesion
MTNR1B	600804	92702789	92715948	11q14.3	Melatonun receptor 1B	Receptor for malatonin, proton-coupled receptors
*C11orf75*	609477	93211638	93276546	11q21	Chromosome 1 open reading frame 75	
TAF1D	612823	93469095	93474703	11q21	TATA box binding protein	Component of the transcription factor SL1/TIF-IB complex. Downregulation induced apoptotic cell death
MED17	603810	93517405	93546496	11q21	Mediator complex subunit 17	Mammalian mediator of transcriptional regulation
PANX1	608420	93862094	93915139	11q21	Pannexin	Structural component of the gap junctions and the hemichannels
*GPR83*	605569	94110477	94134585	11q21	G protein-coupled receptor 83	Orphan receptor
MRE11A	600814	94150466	94227040	11q21	MRE11 meiotic recombination 11 homolog A	Double-strand break repair, DNA recombination, maintenance of telomere integrity and meiosis
FUT4	104230	94277017	94283064	11q21	Fucosyltransferase 4 (alpha (1,3)) myeloid-specific	Biosynthesis of lewis antigene
PIWIL4	610315	94300474	94354587	11q21	Piwi-like 4 (Drosophila)	Development and maintenance of germline stem cells
*KDM4D*	609766	94706845	94732678	11q21	Lysine (K)-specific demethylase 4D	Histone coding
SRSF8	603229	94800056	94804388	11q21	Serine/arginine-rich splicing factor 8	Involved in pre-mrna alternative splicing
SESN3	607768	94906113	94964246	11q21	Sestrin 3	Normal regulation of blood glucose, insulin resistance
CEP57	607951	95523642	95565854	11q21	Centrosomal protein 57 kda	Required for microtubule attachment to centrosomes
*MTMR2*	603557	95566044	95657371	11q21	Myotubularin related protein 2	Tyrosine phosphatase
MAML2	607537	95711440	96076344	11q21	Mastermind-like 2 (Drosphila)	Transcriptional coactivator for NOTCH proteins
JRKL	603211	96123158	96126727	11q21	Jerky homolog-like (mouse)	Not yet defined, probably nuclear regulatory protein

## Discussion

**Fig. 4:**
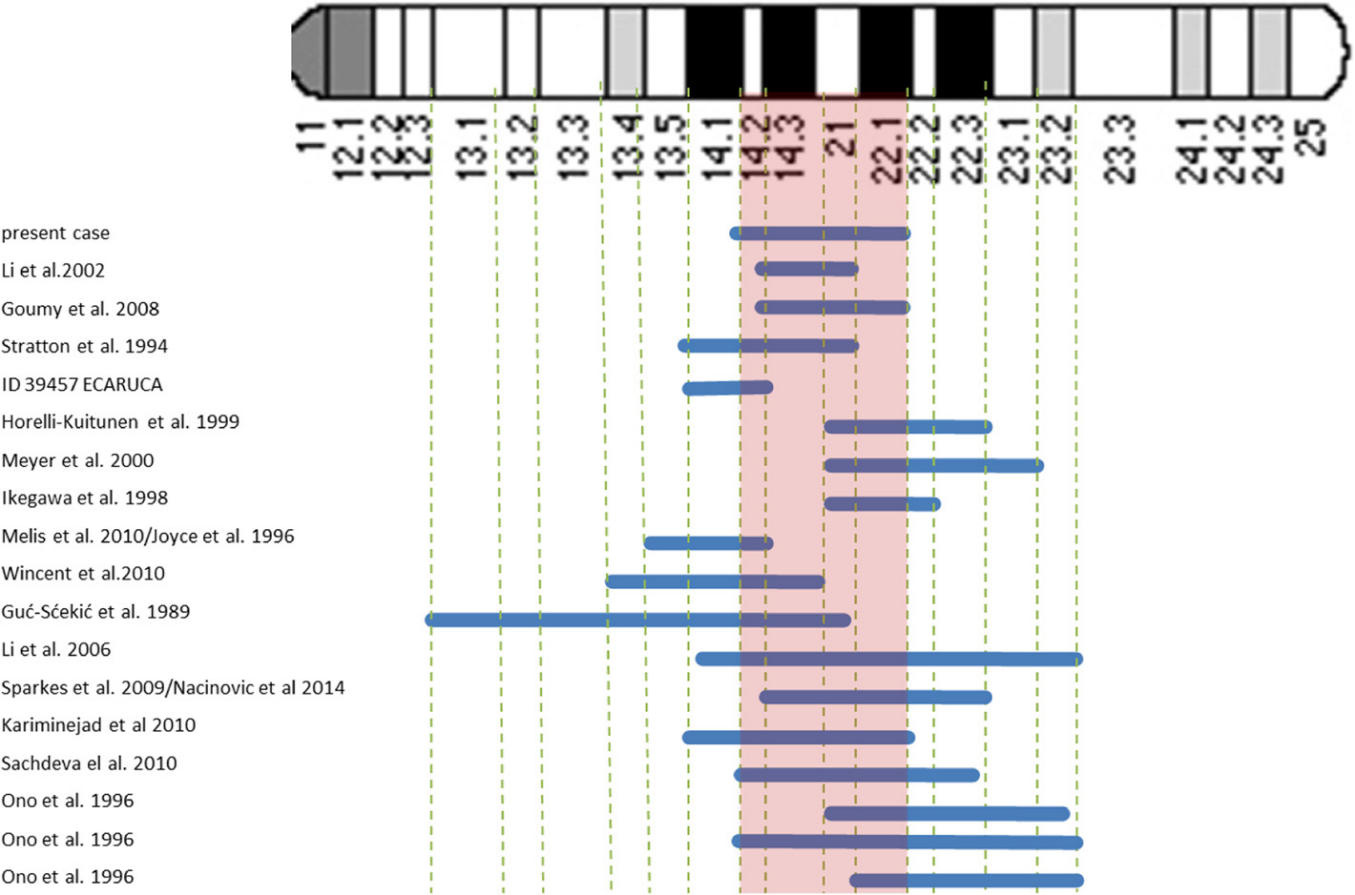
Cases with overlapping interstitial deletions of chromosome 11 long arm. The blue lines show the deletion of each patient, and the pink region illustrates the overlapping region of the present case with the other cases

Here we report a patient with a *de novo* 12.15 Mb interstitial deletion of chromosome 11 long arm, spanning from nt85702633 to nt97854695, exhibiting developmental delay, borderline mental retardation, severe speech delay, and some dysmorphic features. The genotype of the patient was compared to 19 previously described patients carrying overlapping interstitial deletions of chromosome 11 long arm (Fig. 4). Cases with uncertain or not accurately defined breakpoints were not considered [4, 5, 13, 24].

Regarding these 19 cases, only six studies [9–12, 25, 26] were conducted using molecular cytogenetic techniques, such as a-CGH analysis with BAC clones or high-density oligonucleotide probes. In the remaining cases, conventional karyotype analysis and/or fluorescence in situ hybridization analysis was performed in order to define the position and size of the deletions. Table 2 summarizes the genotypes and phenotypic features of the present case and of the 19 cases with overlapping interstitial deletions of chromosome 11 long arm.

**Table 2:**
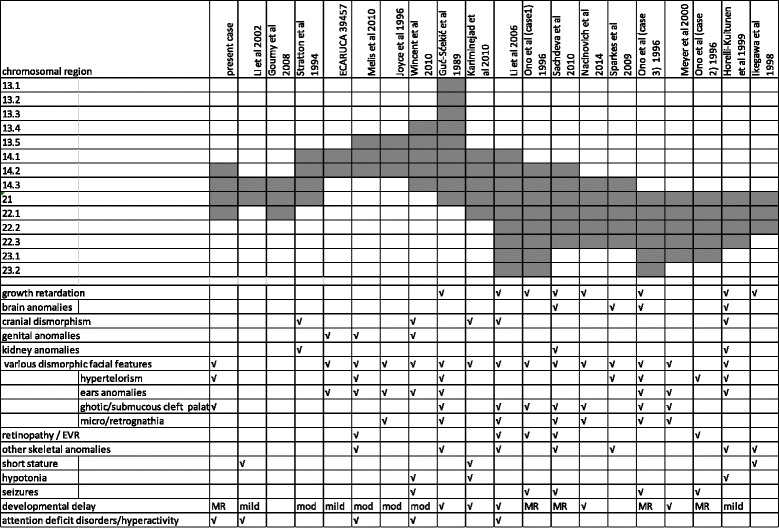
Genotypes and phenotypic features of the present case and of the 19 cases with overlapping interstitial deletions of chromosome 11 long arm

As listed in Table 2, most of the patients with an overlapping deletion of this region had mild to severe developmental delay, short stature/growth delay, high narrow palate or cleft palate/lip with or without migrognathia, and minor digital anomalies. Other clinical features include skeletal anomalies, brain anomalies, cranial dysmorphisms (microcephaly, trigonocephaly), retinal dysgenesis/exudative vitroretinopathy (EVR), genital anomalies, kidney anomalies and heart defects [7, 10, 11, 13, 19]. Regarding the present case with the exception of hypertelorism, and gothic palate our patient had none of these features.

However it is obvious (Table 2) that there are two cases, both without phenotypic abnormalities or developmental delay, which carry similar deletion with the present case. Li et al. [8] described five cases of 11q14.3-q21 deletion transmitted through three-generation kindred. The proband showed short stature and mild attention deficit disorder that required teaching assistant, all other family members were healthy despite carrying the deletion. The deleted region was mapped by FISH with overlapping BAC clones. The entire contig spans 3,6 Mb, and the breakpoints are within clones RP11-792 M23 and RP11-573 M3. In this region there are few genes and only two (MTNBR1 and NAALAD) are single copy genes. Other genes in this region have at least one copy present elsewhere in the genome that might compensate for the deleted copies of these genes.

Goumy et al. [6] described three cases of 11q14.3-q22.1 deletion transmitted in three-generation kindred. The proband, a normal girl without dysmorphic features, was tested during mother’s pregnancy by genetic amniocentesis because of a positive Down syndrome maternal serum screening test at 15 weeks gestation. The deletion was identified in the mother, who had toe camptodactyly and ophthalmologic disorders, and in the phenotipically normal grandfather. The minimal deletion size, mapped by combining CGH analysis and FISH with BAC clones was 8.5 Mb from RP11-372E19 to RP11-775E2. This region contains almost 17 genes in common with those of our case. There might be other genes with similar functions located elsewhere in the genome. Another hypothesis that could explain the lack of phenotypic abnormalities was the haplosufficiency: the adequate functioning of the genes of this region in single copy. Both paternal and maternal origin in the transmission of the deletion, excluded genetic imprinting as explanation of the normal phenotype.

Present case differs from these cases for the presence of 11q14.2 cytogenetic region, exactly from nt 85,668,485 to the first absent BAC clone in case described by Li et al. [8] approximately near the nt 89,255,000. In this region there are genes that should be responsible for the phenotype of this patient. Among the genes contained in the region, GRM5 (Glutamate receptor, metabotropic 5 gene OMIM604102) is particularly interesting. Metabotropic glutamate receptors (mGluRs) are G protein-coupled receptors (GPCRs) which transduce signals from the extracellular matrix to the cytoplasm by activating G proteins*.* One prominent action of group I mGluRs is to protect neurons from apoptotic death [27].

GRM5 plays an important role in modulating neural activity and plasticity [28, 29]. Its signaling is required for different forms of adaptive learning because impaired receptor function results in inappropriate retention of aversive memories, which seems to be related with impaired long-term potentiation in CA1 region and dentate gyrus of the hippocampus [30, 31]. Several neurological and neurodevelopmental disorders are associated with an abnormal function of this gene such as Fragile X syndrome, Schizophrenia anxiety, depression, and addiction [32–34]. All cases which overlaps our case, and in which the deletion includes this gene region, presents developmental delay.

There are four other cases in which developmental delay was present but the region containing this gene was not deleted [16, 19, 20, 25]. However, the region deleted in this four cases includes another Glutamate receptor family gene in 11q22.3 GRIA4 (Glutamate Receptor Ionotrophic Ampa 4) that mediates fast synaptic excitatory neurotransmission (OMIM 138246) and this gene is also implied in neurological disorders in mice [35].

The patients who had normal development had no copy-number variations of both GRM5 and GRIA4 genes [6, 8, 17]. Only in the case described by Sparkes et al. [11], resulted a normal development despite the GRIA4 gene deletion. When the deletions included both genes, there was a more severe phenotype [20, 21]. We hypothesize that both these genes could be associated with developmental delay in 11q interstitial deletions.

The remaining features were impossible to be correlated with any genotype because the deletions in different cases seem to generate many different phenotypes. These chromosomal deletions generally involve a large number of genes, but most of these genes are not dosage sensitive and a single copy of the gene ensures its function. In this situation, when a clinical phenotype was observed, would always be appropriate to analyze the genes on the intact chromosome in order to find mutations. Another mechanism could be the presence of copies or similar genes located elsewhere in the genome that show a compensatory gene expression. Also a deletion within a region subject to genomic maternal or paternal imprinting might not cause pathological phenotype. Therefore further work should be done in animal model organisms in order to fully understand the function of these genes and the pathways that contribute to the phenotype.

## Conclusion

On the basis of current literature we are not yet able to define a monosomy 11q phenotype. Many other studies and accurate molecular characterization are needed to understand the complex genetic and environmental relationship that underlie the phenotypic differences observed in similar cases of chromosomal rearrangements.

## Consent

Written informed consent was obtained from the patient for publication of this Case report and any accompanying images. A copy of the written consent is available for review by the Editor-in-Chief of this journal.

## Abbreviations

ACGH: Array comparative genomic hybridization
HC: Head circumference
CNV: Copy number variation
